# Tension-Tension Fatigue Behavior of High-Toughness Zr_61_Ti_2_Cu_25_Al_12_ Bulk Metallic Glass

**DOI:** 10.3390/ma14112815

**Published:** 2021-05-25

**Authors:** Yu-Hang Yang, Jun Yi, Na Yang, Wen Liang, Hao-Ran Huang, Bo Huang, Yan-Dong Jia, Xi-Lei Bian, Gang Wang

**Affiliations:** Institute of Materials, School of Materials Science and Engineering, Shanghai University, Shanghai 200444, China; yangyuhang@shu.edu.cn (Y.-H.Y.); Yn941129@163.com (N.Y.); liang_wen97@163.com (W.L.); 15249915310@163.com (H.-R.H.); huangb@shu.edu.cn (B.H.); yandongjia@shu.edu.cn (Y.-D.J.); bianxilei@shu.edu.cn (X.-L.B.); g.wang@shu.edu.cn (G.W.)

**Keywords:** bulk metallic glass, fatigue, fracture, striation

## Abstract

Bulk metallic glasses have application potential in engineering structures due to their exceptional strength and fracture toughness. Their fatigue resistance is very important for the application as well. We report the tension-tension fatigue damage behavior of a Zr_61_Ti_2_Cu_25_Al_12_ bulk metallic glass, which has the highest fracture toughness among BMGs. The Zr_61_Ti_2_Cu_25_Al_12_ glass exhibits a tension-tension fatigue endurance limit of 195 MPa, which is higher than that of high-toughness steels. The fracture morphology of the specimens depends on the applied stress amplitude. We found flocks of shear bands, which were perpendicular to the loading direction, on the surface of the fatigue test specimens with stress amplitude higher than the fatigue limit of the glass. The fatigue cracking of the glass initiated from a shear band in a shear band flock. Our work demonstrated that the Zr_61_Ti_2_Cu_25_Al_12_ glass is a competitive structural material and shed light on improving the fatigue resistance of bulk metallic glasses.

## 1. Introduction

Bulk metallic glasses (BMGs) have exceptional strength [1]. For example, Co_43_Fe_20_Ta_5.5_B_31.5_ BMG has the highest strength among bulk metals [2]. The strength of Zr-based BMGs is comparable to that of high-strength steels [3,4]. At the same time, Zr_61_Ti_2_Cu_25_Al_12_ [5] and Pd_79_Ag_3.5_P_6_Si_9.5_Ge_2_ [6] BMGs have high fracture toughness, which is comparable to that of low carbon steels. The combination of the strength and fracture toughness of these two BMGs lie outside the benchmarks established by the strongest and toughest metals [5,6]. Moreover, strain-hardening in Zr_64.13_Cu_15.75_Ni_10.12_Al_10_ has been obtained by using a rejuvenation technique [7]. Therefore, BMGs are now competitive structural material candidates for engineering applications due to their high strength, fracture toughness, and exceptional plasticity. However, fatigue resistance of materials can be vital for engineering structures that are subjected to cyclic loading.

In the early study of metallic glasses in both ribbon and bulk form, their fatigue endurance limits were found to be much lower than that of traditional structural metals [8,9,10,11]. With the advent of many BMGs with new compositions, many research groups have found that a few BMGs have good fatigue resistance. For example, Wang [12] discovered that the fatigue limit of Zr_50_Cu_37_Al_10_Pd_3_ is 983 MPa, which is higher than that of most materials under a tension-tension fatigue test. The Pd content makes the material expensive. This would impede the application of the BMG as an engineering material. A material with high strength, high fracture toughness, high fatigue resistance, and low cost is expected in engineering structures. The combination of the strength and fracture toughness of Zr_61_Ti_2_Cu_25_Al_12_ BMG extends beyond the ranges established by the toughest and strongest materials [5]. At the same time, the cost of the raw material of the BMG is relatively low among BMGs. Therefore, it is of interest to test its fatigue endurance. Four-point bending tests were performed to test its fatigue limit, which is 440 MPa [13]. The fatigue limit of a BMG depends on the testing mode, and the tension-tension fatigue mode is the most destructive [14]. The tension-tension fatigue limit, which is important for the application of the BMG, has not been measured yet. Here, we report the tension-tension fatigue SN curve of the BMG and fracture morphology to investigate its fatigue behavior.

## 2. Materials and Methods

**Figure 1:**
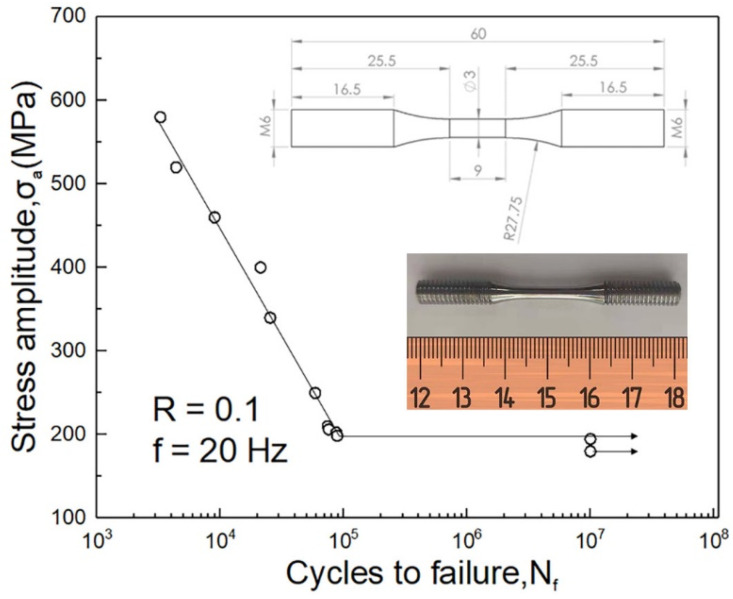
S-N Curve of the Zr_61_Ti_2_Cu_25_Al_12_ glass at an R ratio of 0.1 and a frequency of 20 Hz. The fatigue limit of the glass is 195 MPa. The insets show the mechanical drawing and a photograph of the test specimens.

Zr_61_Ti_2_Cu_25_Al_12_ rods with a diameter of 6 mm and a length of 80 mm were prepared by arc-melting elements with a purity higher than 99.95% and subsequent suction casting in a vacuum chamber (Beijing, China) with Ti-gettered pure argon atmosphere. Details of the fabrication procedure and confirmation of their fully amorphous nature can be found in our previous work [15]. Rods with a length of 60 mm were cut from the bottom of the as-cast rods by using a diamond wafering blade. The tension-tension fatigue test cylindrical specimens with tangentially blended fillets between the test section and the grip section were carefully machined from the rods according to ASTM-E466-07 standard. The radius of the fillets was 27.75 mm. The length and diameter of the gauge section were 9 mm and 3 mm, respectively. The grip section was machined with M6 threads. The gauge section was carefully polished by using strips of SiC paper that were wound around rotating fatigue test specimens. The mechanical drawing and a photograph of the fatigue test specimens are shown in the insets of Figure 1. The tension-tension fatigue tests were performed on an Instron 8801 Servohydraulic Dynamic Testing System (Norwood, MA, USA) with an R ratio of 0.1 and a frequency of 20 Hz at room temperature. In order to prevent the generation of fatigue crack between threads on the grip section, thread seal tape was applied onto the threads before mounting the test specimens onto the fatigue test fixture. The morphology of the fractured specimens was examined in a Hitachi SU-1510 scanning electron microscope (SEM) (Tokyo, Japan).

## 3. Results

### 3.1. S-N Curve

The dependence of the number of cycles to failure (N_f_) on cyclic tensile stress amplitude $\sigma_{a}=\left(\sigma_{\max}-\sigma_{\min}\right)/2$ (σ_max_ and σ_min_ is the maximum and the minimum stress, respectively) for the Zr_61_Ti_2_Cu_25_Al_12_ BMG is plotted in Figure 1. The tension-tension S-N curve with a shape that is very similar to steels and polymers [16] shows that the BMG has a distinct fatigue limit. At a $\sigma_{a}$ of 198.75 MPa, the fatigue life of the BMG is 88,452 cycles while it increases to more than or equal to 10^7^ cycles with a 195 MPa $\sigma_{a}$ as shown in Figure 1. The distinctness demonstrates that no defect that initiates a fatigue crack can be produced by stress amplitude lower than the fatigue limit and promises safe application of the BMG in cyclic loading conditions. The fatigue limit is much lower than that of the Zr_61_Ti_2_Cu_25_Al_12_ BMG under the four-point bending fatigue test, which is 441 MPa [13]. The tensile stress decreases from the maximum on the bottom surface of the four-point bending specimen to zero on its neutral plane, while the tensile stress is homogeneous in the tensile specimen. Therefore, a crack is much more easily opened under uniaxial tension than under four-point bending [17]. Moreover, it has been proven that a fatigue crack initiates an open shear band [18]. We believe that this is the reason why the tension-tension fatigue limit is lower than the four-point bending fatigue limit. Nevertheless, the tension-tension fatigue limit of the Zr_61_Ti_2_Cu_25_Al_12_ BMG is higher than steels (of which fatigue limit is around 150 MPa) that are applied in the automotive industry with comparable fracture toughness [19]. In addition, the Zr_61_Ti_2_Cu_25_Al_12_ BMG fractures elastically in tension, and its tensile strength and Young’s modulus are 1672 MPa and 82.8 GPa, respectively. From the perspective of the combination of strength, fracture toughness, and fatigue limit, we believe that the Zr_61_Ti_2_Cu_25_Al_12_ BMG is a competitive candidate material for engineering structures that are subjected to cyclic loading.

### 3.2. Fracture Morphology

**Figure 2:**
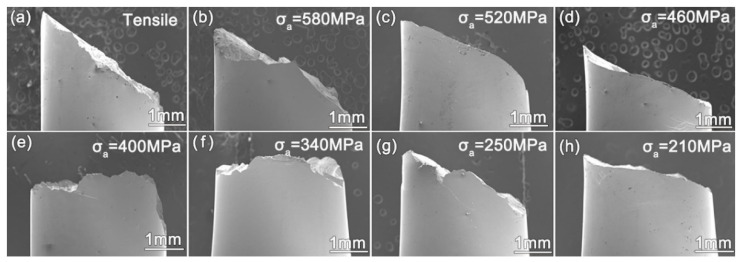
(**a**) Fractured Zr_61_Ti_2_Cu_25_Al_12_ glass specimen in uniaxial tension. (**b**–**h**) Side morphology of Zr_61_Ti_2_Cu_25_Al_12_ specimens fatigue fractured at tensile stress amplitudes of 580, 520, 460, 400, 340, 250, and 210 MPa, respectively.

In order to further investigate the fatigue behavior of the Zr_61_Ti_2_Cu_25_Al_12_ BMG, SEM was used to image the specimen-free surface and also the crack surface morphology of fractured specimens after applications of various $\sigma_{a}$. The SEM images of the side surface morphology are shown in Figure 2. Figure 2a shows a shear fractured specimen under quasi-static tension. The fracture angle is 49.6°. The catastrophic fracture angle of the specimens shown in Figure 2b–h decreases with decreasing $\sigma_{a}$. On the other hand, the length of the fatigue crack before static fracture increases with decreasing $\sigma_{a}$ [20]. At the same time, all the samples should catastrophically fracture at the same critical stress intensity factor [21], and the normal stress for opening a shear band in the plastic zone increases with decreasing $\sigma_{a}$. This explains why the catastrophic fracture angle of the specimens decreases with decreasing $\sigma_{a}$.

**Figure 3:**
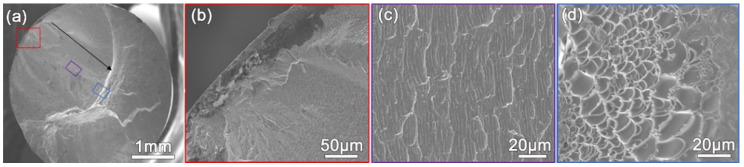
Fracture surface of the Zr_61_Ti_2_Cu_25_Al_12_ BMG tested at the stress amplitude of 460 MPa. (**a**) Overview of the fracture surface. The black arrow shows the direction of fatigue crack propagation. Details in the red, purple, and blue box are shown in (**b**–**d**), respectively. (**b**) Initiation site of the fatigue crack. (**c**) Fatigue striations formed during fatigue crack propagation. (**d**) Dimples generated during catastrophic fracture.

The fracture surface morphology of the specimen fractured under a $\sigma_{a}$ of 460 MPa is shown in Figure 3. The overview of the fracture surface in Figure 3a shows the fatigue crack initiation site, fatigue crack growth region, and catastrophic fracture region. The shear offset and fatigue initiation site in Figure 3b demonstrate that the fatigue crack initiated from a shear band because the shear offset indicates shear banding in BMGs as reported in the literature [22]. The coarse and fine fatigue striations in Figure 3c were imaged in the fatigue crack growth region in Figure 3a. A fatigue crack propagates along shear bands in BMGs, and one coarse striation corresponds to one shear band segment [18]. At the same time, a fine striation corresponds to the advance of the fatigue crack during one loading cycle [18]. The average length of the fine striations in Figure 3c was determined to be 38.6 μm. We deduced that the average dimension of shear bands ahead of the fatigue crack tip in the direction of specimen thickness would be about 38.6 μm. Therefore, the coarse striations in Figure 3c show the morphology of shear band segments in the plastic zone ahead of a fatigue crack. The viscous vein pattern in Figure 3d indicates the final catastrophic fracture of the specimen.

### 3.3. Morphology of Shear Bands

**Figure 4:**
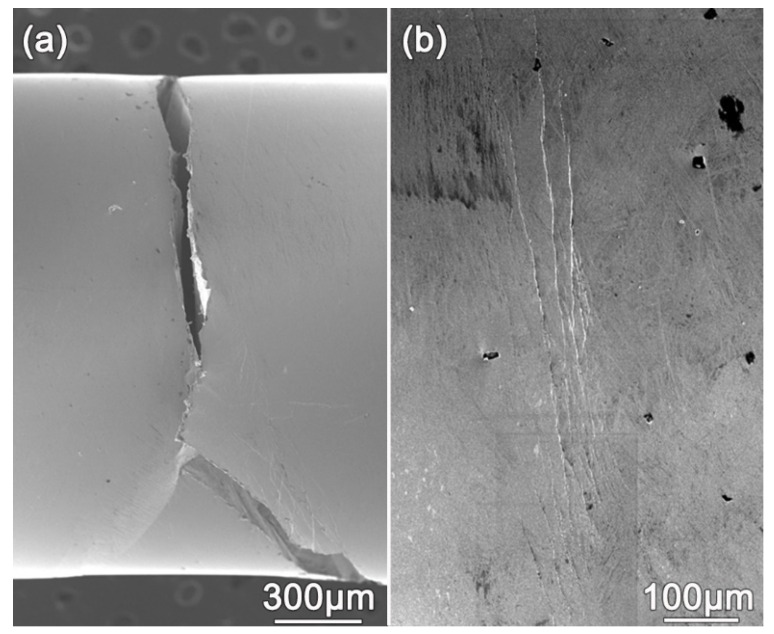
Fatigue crack initiation shown on the side surface of fractured Zr_61_Ti_2_Cu_25_Al_12_ BMG specimen tested at the stress amplitude of 460 MPa. (**a**) The SEM image shows that the fatigue crack initiated at a shear band. (**b**) A flock of shear bands on the side surface of the specimen far away from the fracture surface.

The side surface morphology of the specimen fractured at the $\sigma_{a}$ of 460 MPa is shown in Figure 4. Figure 4a indicates that fatigue crack initiated at a shear band in a shear band flock. As reported [23], the shear bands shown in Figure 4a formed at the stress level much lower than quasi-static yield strength because of softening induced by cyclic loading. At positions far away from the crack, flocks of shear bands were found. Figure 4b shows a shear band flock. The shear bands were perpendicular to the loading direction. The shear offset shown in Figure 3b also demonstrates the direction of the shear bands. At the same time, on the side surface of the specimens tested at $\sigma_{a}$ below the fatigue limit, no shear band was found. This phenomenon indicates that the fatigue limit under the tension-tension mode would be the maximum $\sigma_{a}$ under which no shear band could form. Therefore, the low tension-tension fatigue limit of the Zr_61_Ti_2_Cu_25_Al_12_ BMG might be caused by the ease of shear band formation.

## 4. Discussion

Our previous results [15] demonstrated that the content of Hf in the Zr_61_Ti_2_Cu_25_Al_12_ BMG is lower than that of the BMG with the same nominal composition in the reference [24]. The Allen electronegativity of Hf element is the smallest among the elements in the Zr_61_Ti_2_Cu_25_Al_12_ BMG. The Hf in the glass tends to form strong covalent bonding with Al to reduce free volume. Therefore, the free volume could be increased by reducing Hf content in the BMG. The high fracture toughness of the BMG [15] indicates higher free volume content. Even though it has been proven that change in the free volume cannot affect fatigue crack growth in BMG, the change could affect fatigue initiation and thus fatigue life and fatigue limit [25]. The fatigue limit of BMGs can be significantly improved by annealing, which induces free volume reduction [25,26]. The reduction in Hf content would be the reason for the relatively low tension-tension fatigue limit of the specimens among BMGs. Nevertheless, the fatigue limit of the Zr_61_Ti_2_Cu_25_Al_12_ BMG is still higher than many tough steels. There is a trade-off between the fatigue resistance and the fracture toughness of BMGs [26]. As far as we know, no technique has been developed to eliminate the trade-off. We think the reason is that the mechanism of fatigue crack propagation is still elusive. Many experiments have proven that the microstructure of metallic glass is topologically heterogeneous [27,28,29]. The heterogeneity strongly affects the deformation [30] and fracture [31] behavior of metallic glasses. At the same time, shear bands in BMGs have been found to be chemically heterogeneous by using atom probe tomography [32,33]. As reported in the literature [34], the fatigue crack propagated along the shear bands. In addition, heterogeneity-related soft spots with large free volume can result in catastrophic fracture. Therefore, we believe that the chemical heterogeneity in shear bands can strongly affect the propagation of a fatigue crack in BMGs. As far as we know, the formation mechanism of the fine fatigue striations, as shown in Figure 3c, has not been revealed fundamentally until now. Therefore, physical models taking into account the chemical heterogeneity of shear bands need to be undertaken to elucidate the fatigue behavior of BMGs.

## 5. Conclusions

In summary, the tension-tension fatigue behavior of Zr_61_Ti_2_Cu_25_Al_12_ BMG with the highest fracture toughness among BMGs was investigated. Its tension-tension fatigue limit is higher than many engineering structural steels, and this makes it a competitive structural material candidate. Fracture morphology investigation indicated that the fatigue crack was induced by flocks of shear bands with a direction perpendicular to the loading direction because the fatigue crack initiated from one of the shear bands. The present work indicates that the fatigue resistance of BMGs could be improved by suppressing the formation of such shear bands.

## Data Availability

Data are available upon request to the corresponding author.
